# Navitoclax acts synergistically with irradiation to induce apoptosis in preclinical models of H3K27M-altered diffuse midline glioma

**DOI:** 10.1038/s41598-025-32676-6

**Published:** 2025-12-22

**Authors:** Ashley Vardon, Scott Haston, Hiba Hamdi, Grace Cooksley, Romain Guiho, Diana Carvalho, Rebecca Carter, Jessica Katherine Rowena Boult, Daniel Gharai, Ielyaas Cloete, James Grey, John Apps, Daohong Zhou, Guangrong Zheng, Ramón Martínez-Máñez, Mark Francis Lythgoe, Laura Kate Donovan, Angel Montero Carcaboso, Owen Williams, Ying Hong, Paula Alexandre, Jesus Gil, Jamie Adam Dean, David Michod, Chris Jones, Darren Hargrave, Juan-Pedro Martinez-Barbera

**Affiliations:** 1 Developmental Biology and Cancer Programme, Birth Defects Research Centre, Great Ormond Street Institute of Child Health, University College London, London, UK; 2 Brain Tumour Research Centre of Excellence, Division of Molecular Pathology, Institute of Cancer Research, London, UK; 3 UCL Cancer Institute, University College London, Paul O’Gorman Building, London, UK; 4 Division of Radiotherapy and Imaging, Institute of Cancer Research, London, UK; 5 Department of Medical Physics and Biomedical Engineering, University College London, London, UK; 6 Department of Biochemistry and Structural Biology and Center for Innovative Drug Discovery, University of Texas Health San Antonio, San Antonio, USA; 7 University of Florida Health Cancer Center, University of Florida, Gainesville, USA; 8 Instituto Interuniversitario de Investigación de Reconocimiento Molecular y Desarrollo Tecnológico (IDM), Universitat Politècnica de València, Universitat de València, València, Spain; 9 CIBER de Bioingeniería, Biomateriales y Nanomedicina (CIBER-BBN), Instituto de Salud Carlos III, Madrid, Spain; 10 SJD Pediatric Cancer Center Barcelona, Hospital Sant Joan de Deu, Institut de Recerca Sant Joan de Deu (IRSJD), Barcelona, Spain; 11 Infection, Immunity and Inflammation Programme, UCL Great Ormond Street Institute of Child Health, University College London, London, UK; 12 MRC Laboratory of Medical Sciences (LMS), Du Cane Road, London, W12 0NN UK; 13 Institute of Clinical Sciences (ICS), Faculty of Medicine, Imperial College London, Du Cane Road, London, W12 0NN UK; 14 Physics of Living Systems, University College London, London, UK; 15 Present Address: Institute of Cancer and Genomic Medicine, University of Birmingham, Birmingham, UK; 16 Present Address: INSERM, Regenerative Medicine and Skeleton, Nantes Université, Oniris, RMeS, UMR 1229, F-44000 Nantes, France

**Keywords:** H327M-altered diffuse midline glioma, Cellular senescence, Senolytic therapy, BH3-mimetics, Bcl-xL, Cancer, Paediatric cancer, Senescence

## Abstract

Diffuse midline gliomas (DMGs) with histone H3K27M mutations represent a devastating paediatric brain cancer characterised by abysmal prognosis and limited treatment options. The only approved treatment is radiotherapy (RT), but most of the tumours relapse with fatal consequences. The effects of RT remain unknown because patients are not biopsied during treatment. Here, we sought to investigate whether irradiation leads to senescence induction in DMG and explore the efficacy of senolytics. We show that ionising radiation induces senescence in various H3K27M-altered DMG cell lines. Senescence induction is demonstrated by immunocytochemistry, RNA-sequencing and analysis of SASP factors by ELISA. Through testing several senolytic compounds, we identify that Bcl2 family inhibitors (e.g., Navitoclax) act as potent senolytics, driving senescent DMG cells into apoptosis, primarily via Bcl-xL inhibition. Reinforcing these findings, proteolysis-targeting chimeras (PROTACs) targeting Bcl-xL and galacto-conjugated Navitoclax (Nav-Gal) also exhibit strong senolytic activity against senescent DMG cancer cells. Finally, we show that a combination of irradiation with Navitoclax enhances cancer cell apoptosis in an orthotopic xenograft DMG model. Together, the data demonstrate that ionising irradiation leads to senescence induction in H3K27M-altered human DMG cell lines, making them particularly sensitive to apoptosis through Bcl-xL inhibition.

**Supplementary Information:**

The online version contains supplementary material available at 10.1038/s41598-025-32676-6.

## Introduction

H3K27M-altered diffuse midline glioma (DMG) of the pons represents around 10% of childhood brain tumours, and has a peak incidence of 6–8 years. Despite four decades of clinical trials, the only proven effective therapy is radiotherapy (RT), and unfortunately this is palliative and most patients relapse and succumb to the disease with a median survival of 8–11 months post-diagnosis^1^. RT can induce cellular senescence in a variety of cancer contexts including brain tumours^2–5^. While senescence induction contributes to the initial response to RT, lingering senescent cells within the irradiated tumour bed may contribute to create a permissive environment that promotes relapse. Therefore, targeting of these senescent cells may represent a novel therapeutic strategy for combating DMG.

Cellular senescence is characterised by stable cell cycle arrest, which is maintained by critical pathways regulating cell cycle progression (e.g. p53/p21^CIP^^1^ and p16^INK4a^/RB)^6^. Senescent cells can elicit cell non-autonomous activities through the Senescence-Associated Secretory Phenotype (SASP), a complex secretory programme composed of a multitude of cytokines and chemokines (e.g. IL1α, IL1β, IL6), growth factors (e.g. EGF, FGFs, VEGF), and other biomolecules^7^. Although SASP induction can trigger an immune surveillance response contributing to tumour clearance, chronic SASP activation can promote proliferation of transformed cells and/or create a permissive microenvironment that supports tumour progression, malignancy and metastasis^8^. Of clinical relevance, senescent cell ablation or modulation of the SASP, can reduce tumour burden, increase mouse survival, decrease tumour relapse and alleviate the negative effects of anticancer treatment^9,10^.

Senescent cells can be ablated effectively using senolytic compounds, which exploit molecular vulnerabilities in senescent cells. Several drugs that can selectively kill senescent cells have been identified, including dasatinib and quercetin (referred to as D + Q)^11^, Bcl2 family inhibitors such as ABT-263 (also known as Navitoclax) and ABT-737^12,13^, a modified FOXO4-p53 interfering peptide^14^, HSP90 inhibitors (e.g., alvespimycin)^15^, piperlongumine^16^, cardiac glycosides^17,18^, N-myristoylation inhibitors^19^ and β-galactosidase-activated nanoparticles and pro-drugs^20–22^. Among all senolytics, inhibition of anti-apoptotic Bcl2 family proteins with BH3-mimetics is one of the most commonly used methods to eliminate senescent cells pharmacologically both in vitro and in vivo across a variety of cell types^23–27^.

In this study, we have characterised the effects of radiation regimens on a variety of molecularly distinct H3K27M-altered human DMG cellular models, demonstrating the induction of a senescence programme and a vulnerability of RT-induced senescent DMG cells to Bcl-xL inhibition.

## Results

### H3K27M-altered DMG cancer cell lines undergo senescence induction when exposed to ionising radiation

**Fig. 1:**
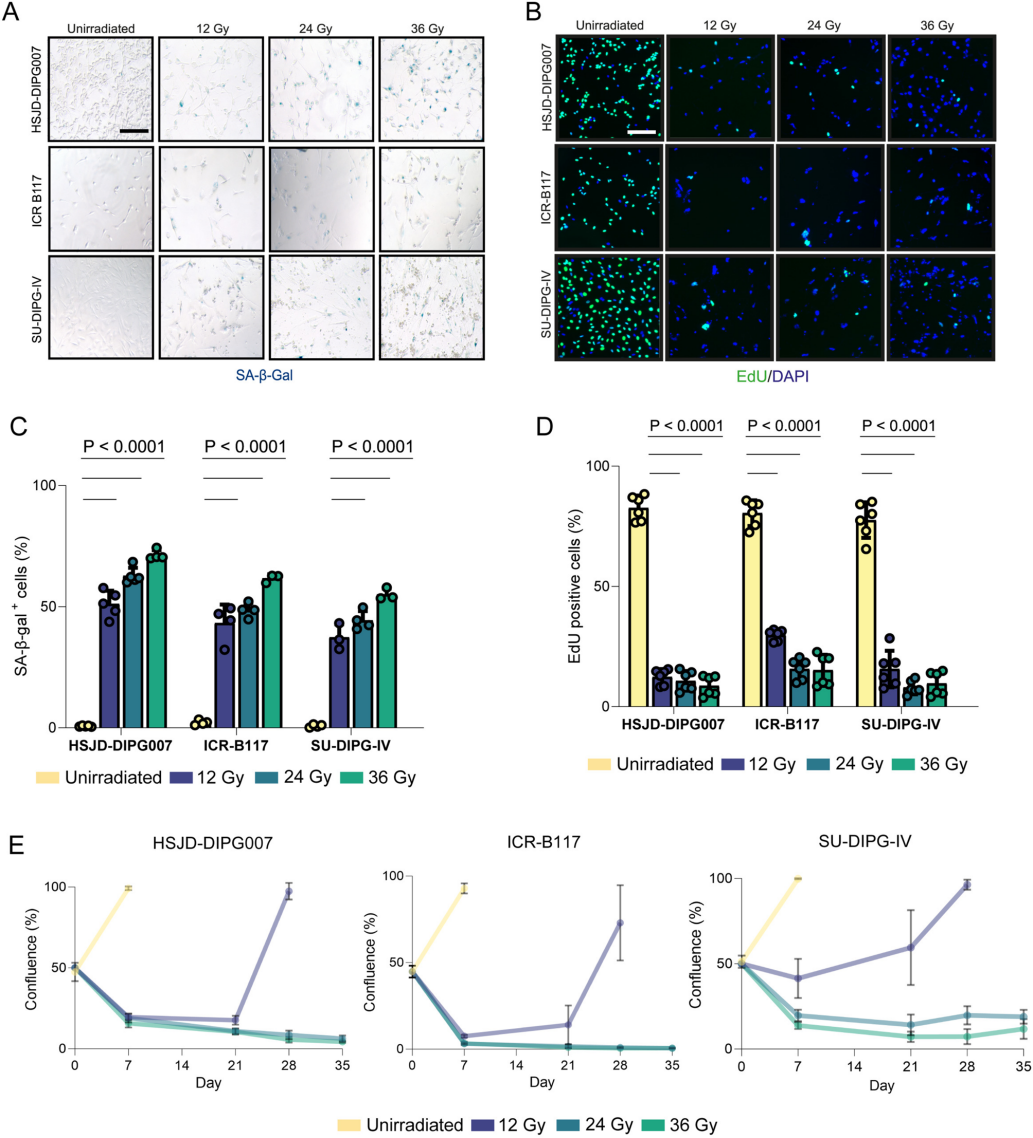
Following irradiation H3K27M-DMG cells exhibit growth arrest and express markers of senescence. (**A**) Representative images of SA-β-gal staining of unirradiated control and irradiated DMG cells after different doses of radiation. (**B**) Representative images of EdU staining of unirradiated control and irradiated DMG cells after different doses of radiation. (**C**) Quantification of SA-β-gal positive cells from experiments shown in B. (**D**) Quantification of EdU positive cells from experiments shown in C. (**E**) Cell confluency of DMG cell lines either unirradiated or treated with single doses of radiation (12, 24, 36 Gy). Irradiated cells were monitored over a 35-day period. Error bar mean ± SD. Colour relating to treatment. Legend colour representation of doses of radiation used. One-way ANOVA. Scale bar = 50 μM.

We first assessed whether H3K27M-altered DMG cell lines of different genotypes (Supplementary Table 1) could be driven into senescence by irradiation. ICR-B117, HSJD-DIPG007 and SU-DIPG-IV cells were exposed to single doses of 12, 24 or 36 Gy and analysed 5 days later for senescence-associated β-galactosidase (SA-β-Gal) staining and EdU incorporation, as markers of senescence and proliferation, respectively. Irradiation induced a marked, dose- and cell line-dependent increase in SA-β-Gal-positive cells relative to non-irradiated controls, which showed negligible staining (Fig. 1A,C). Across the three lines, SA-β-Gal-positive cells constituted ~ 30–70% of DAPI-positive cells depending on dose (Fig. 1A,C). This induction of SA-β-Gal was accompanied by a substantial reduction in EdU incorporation: all irradiated cultures exhibited < 12% EdU-positive cells, with the exception of ICR-B117 at 12 Gy, which retained ~ 25% EdU incorporation (Fig. 1B,D). In contrast, non-irradiated cells showed > 80% EdU incorporation.

Lower doses (4 and 8 Gy) also triggered SA-β-Gal positivity and transient cell-cycle arrest in DIPG-IV and DIPG007 (Supplementary Fig. 1). However, only the higher 24 Gy and 36 Gy doses produced a durable proliferative arrest that was maintained throughout the 35-day observation period. Crucially, irradiation doses of 12 Gy or below failed to establish a stable senescent state: although these doses elicited early SA-β-Gal induction, cultures consistently re-entered the cell cycle within 2–3 weeks post-irradiation (Fig. 1E). As a result, doses ≤ 12 Gy were insufficient for generating a sustained senescent population suitable for downstream senolytic testing.

DMG patients are treated with 54 Gy in 30 fractions of 1.8 Gy^28^. To attempt to mirror this more clinically applicable fractionation schedule, we exposed H3K27M-altered DMG cancer cells to 24 Gy, in 12 fractions of 2 Gy (Supplementary Fig. 2**A**). Analysis of cells 24 h after the final irradiation dose showed that this regimen elicited a response similar to that observed following a single exposure, characterized by increased SA-β-Gal staining and reduced EdU incorporation compared with non-irradiated controls (Supplementary Fig. 2**B, C**). Moreover, qRT-PCR analysis revealed that cell irradiation led to the upregulation of *CDKN1A* (encoding p21) expression and down-regulation of *LMNB1*, encoding Lamin B1, a component of the nuclear lamina that is downregulated in senescent cells in a variety of cell contexts^29^ (Supplementary Fig. 2**D).** However, this regimen was less practical for experimental work due to logistical constraints. Therefore, we continued the study using single irradiation doses, specifically 24 Gy, as this dose produced a stable cell cycle arrest and a clear senescent phenotype. Collectively, these data indicate that both single and fractionated irradiation can induce a senescence response in DMG cancer cells, although the duration of this response is dependent on the cell type and irradiation dose.

**Fig. 2:**
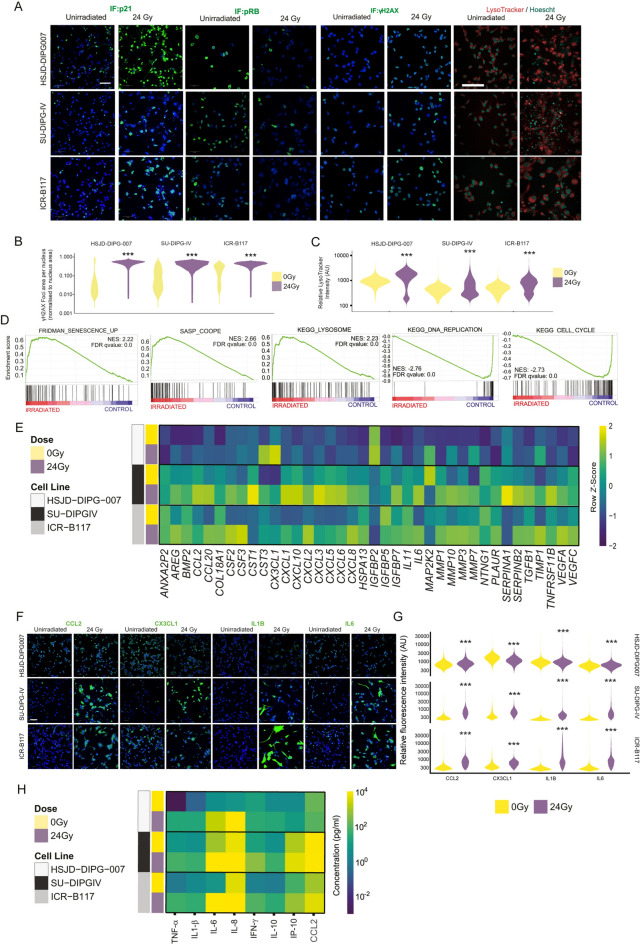
Following irradiation H3K27M-DMG cells exhibit a robust senescence phenotype (**A**) Immunofluorescence (IF) staining of p21 (CDKN1A), phospho-RB and γH2AX, and red-fluorescent dye LysoTracker live cell staining in three DMG cell lines following a single dose of irradiation (24 Gy). Scale bar = 100 µM (**B**, **C**) Quantification of the γH2AX and LysoTracker staining shown in A. Fluorescence intensity is indicated in arbitrary units (AU). (**D**) Gene Set Enrichment Analyses (GSEA) showing a significant enrichment for the FRIDMAN_SENESCENCE_UP, SASP_COOPE and KEGG_LYSOSOME gene sets in irradiated DMG versus unirradiated control cells. Note that the KEGG_DNA_REPLICATION and CELL_CYCLE gene sets are enriched in unirradiated control cells. (**E**) Heatmap of RNA sequencing analysis of DMG cell lines following 24 Gy of radiation revealing an upregulation of genes associated with senescence/SASP. (**F**) Immunofluorescence staining of the SASP factors: CCL2, CX3CL1, IL1B and IL6 in DMG cell lines following irradiation (24 Gy). (**G**) Quantification of fluorescence intensity (arbitrary units, AU) is plotted for each cell line. (**H**) Meso-scale discovery cytokine quantification was carried out on conditioned media 5 days following irradiation. Heatmap represents log fold change cytokine concentration (pg/ml). One-way ANOVA. NES = normalised enrichment score. FDR = false discovery rate.

To characterise further this potential senescent phenotype, we used a combination of biochemical and cytological staining techniques. Relative to unirradiated control cells, irradiated DMG cell lines displayed increased expression of the cyclin-dependent kinase inhibitors, p21, p27 (encoded by the *CDKN1B*) and p57 (encoded by the *CDKN1C*) following immunofluorescence analysis (Fig. 2A, B** and **Supplementary Fig. 3**A, B**). We did not analyse p16 (encoded by *CDKN2A*) expression because this locus is repressed in DMG cells harbouring K27M mutations in the H3.3 (encoded by the *H3F3A* gene)^30^. Conversely, we observed decreased phosphorylation of the Rb protein (pRB), an important regulator of cell cycle progression, in irradiated relative to non-irradiated control cells **(**Fig. 2A). Irradiation is expected to cause DNA damage, which is a potent senescence inducer^31^. γH2AX immunofluorescence staining, which detects double DNA breaks^32^, revealed a significant increase in its expression in irradiated relative to non-irradiated control cells (Fig. 2A, B). Moreover, CRYAB expression, a member of the small heat shock family of proteins and a suggested marker of cellular senescence^33^, was also upregulated in irradiated DMG cells (Supplementary Fig. 3C, D). Another key feature of senescent cells is the enlargement of the lysosomal compartment^34^. Staining of the cells with Lysotracker, a red-fluorescent dye that labels lysosomes, revealed a significant increase in fluorescence intensity in irradiated relative to non-irradiated cells, indicating increased lysosome mass (Fig. 2A, C; Supplementary Methods). Senescence induction upon irradiation was also demonstrated in an additional DMG cell line, HSJD-DIPG14A (Supplementary Fig. 4). Together, these analyses suggest that irradiation leads to a senescent phenotype in DMG cell lines.

To complement our cytological analyses with a molecular characterisation, we performed bulk RNA-sequencing on ICR-B117, SU-DIPG-IV and HSJD-DIPG007 cell lines prior to and after irradiation (single dose of 24 Gy, day 5 post-irradiation). Computational analysis combining the datasets from the three DMG cell lines revealed that a total of 2153 genes were significantly differentially expressed between the irradiated and non-irradiated cell lines (adjusted P value ≤ 0.1, Fold Change ≥  ± 2), comprising 1461 upregulated genes and 692 downregulated genes (Supplementary Fig. 3E, F and Supplementary Table 5). Among the significantly upregulated genes in irradiated cells, we found a number of genes relevant to the senescent phenotype: (i) cell cycle inhibitors (e.g. *CDKN1A* (encoding p21, Fold change 3.2, adjusted p-value < 0.0001); (ii) lysosomal genes (e.g. *GLB1, MAN2B1, HEXA*; Fold change 1.5, 2.3, twofold respectively, adjusted p-value all < 0.1); (iii) SASP factors (e.g. *MMP3* (Fold change: 98, adjusted p < 0.0001), *IL1B* (Fold change: 311), *CXCL8* (Fold change: 267) *CX3CL1* (Fold change 34) and *FGF7* (Fold change: 85) (all adjusted p-values < 0.0001). Importantly, in corroboration with our qRT-PCR and immunofluorescence data we did not observe an up regulation in *CDKN2A* (Fold change 1.27, adjusted value p = 0.6)*.* In agreement with a senescence phenotype, genes normally associated with proliferation, such as *E2F2, MYBL2,* and *ZWINT* were downregulated in irradiated cells (Fold change 6, 6, 5 respectively, adjusted p-value < 0.0001), as well as *LMNB1* (Fold change 4.7, p-value < 0.0001)) (Supplementary Fig. 3E-H; Supplementary Table 5).

Gene set enrichment analysis (GSEA) revealed a significant enrichment for senescence^35^, SASP^36^ and KEGG Lysosome signatures in irradiated compared with non-irradiated control cells (Fig. 2D). Conversely, gene expression signatures associated with cell cycle progression were all significantly enriched in non-irradiated control cells **(**Fig. 2D). GSEA of the Hallmark gene set collection (from the Molecular Signatures Database, MSigDB) revealed enrichment of stress and inflammation related processes (i.e., Hypoxia, IL6-JAK-STAT3 signalling, inflammatory response and TNFα signalling) in irradiated DMG cells (Supplementary Fig. 3I) and proliferative responses (i.e., mitotic spindle, G2M checkpoint and E2F targets) in the unirradiated control DMG cells (Supplementary Fig. 3 J). In agreement, gene ontology (GO) analysis revealed similar stress and inflammatory processes in irradiated cells (Supplementary Fig. 3 K) and proliferative responses in unirradiated cells (Supplementary Fig. 3L).

From our transcriptomic analysis we also observed upregulation of a number of SASP factors in irradiated DMG cell lines (Fig. 2E**)**. To validate these findings, we performed immunofluorescence staining against CCL2, CX3CL1, IL1β and IL6, in either irradiated or unirradiated control DMG cells, revealing the increased expression in the irradiated group (Fig. 2F, G). Moreover, ELISA analysis of conditioned medium collected on day 5 post-irradiation demonstrated the increased secretion of several senescence-associated cytokines and chemokines in irradiated DMG cells relative to control medium, including the prominent SASP factors IL1β, TNFα, IL6 and CCL2 (Fig. 2H). Importantly, clinically relevant fractionated doses of ionizing radiation were also sufficient to induce upregulation of the SASP markers IL1B and CCL2, relative to non-irradiated proliferative control cells (Supplementary Fig. 2**D**). Collectively, these molecular and cellular analyses demonstrate that ionising irradiation induces DNA damage, reduces proliferation, triggers senescence-associated markers, and activates a secretory phenotype in H3K27M-altered human DMG cell lines.

### Navitoclax is a potent senolytic in H3K27M-altered human DMG cell lines

We next aimed to explore possible therapeutic implications of leveraging the senescent phenotype through the use of senotherapies to ablate senescent DMG cells. We tested four described senolytic agents, Dasatinib plus Quercetin (D + Q), Piperlongumine, Alvespimycin and Navitoclax, on senescent (irradiated) and control proliferative (unirradiated) DMG cells.

**Fig. 3:**
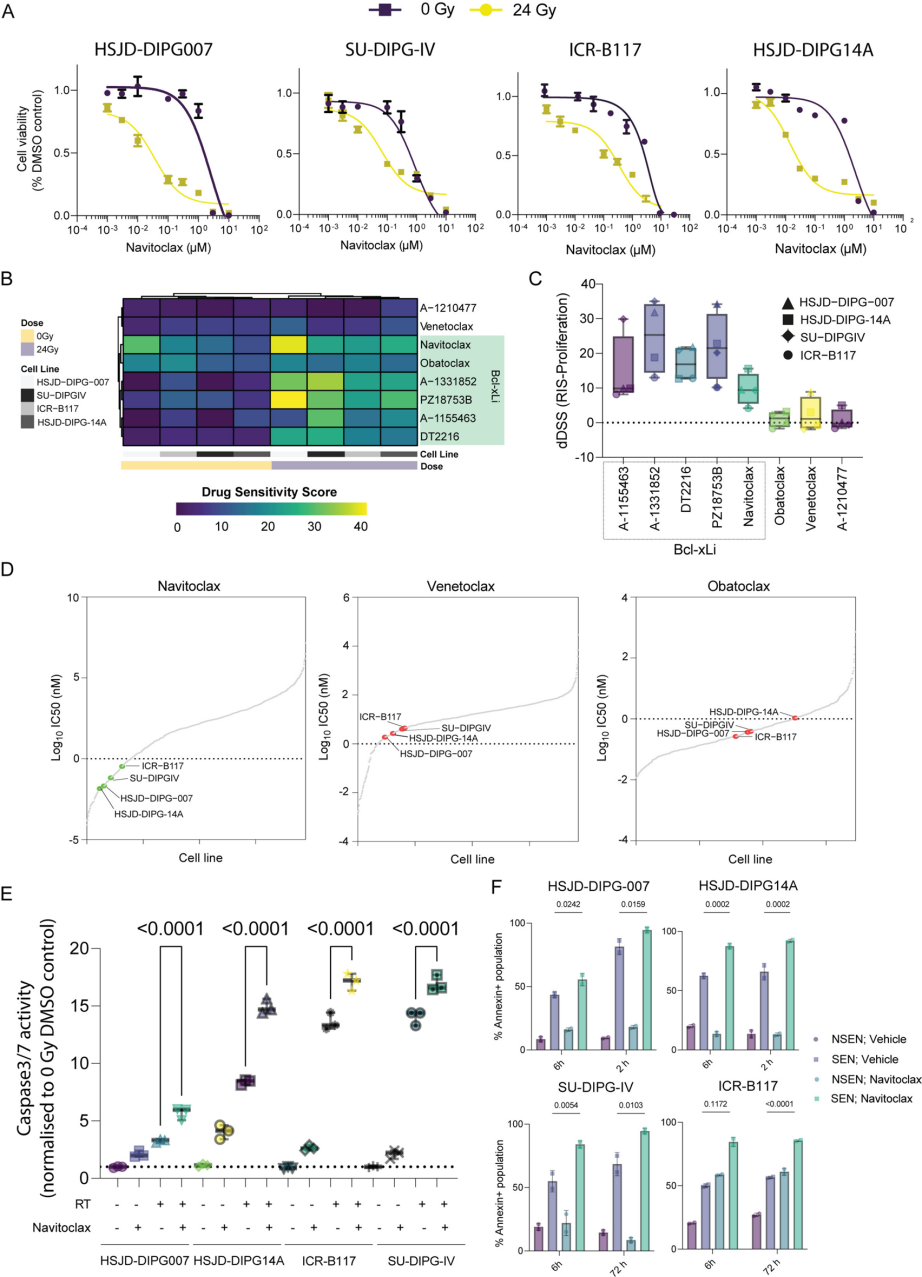
Bcl-xL inhibition is a targetable vulnerability in H3K27M-DMG cells following irradiation. (**A**) Dose–response curves of four human DMG cell lines exposed to a range of Navitoclax concentrations in senescent (yellow) or proliferating (purple) states. Values are shown are mean ± SD of three technical replicates. (**B**) Drug sensitivity scores were calculated based on dose–response data (n = 3 biological replicates for each drug, cell line and state) using Breeze (version 2.0) software. (**C**) Dotplot depicting mean differential drug sensitivity scores (dDSS) of the indicated drugs across four DMG cell lines (mean ± SD). dDDS are calculated by subtracting DSS of proliferating cells from DSS of senescent cells. (**D**) Ranking of the IC50 values for BH3 mimetic sensitivity in the four senescent DMG cell lines against 967 cell lines from the GDSC database. The sensitivity line is set at 1 μM. (**E**) The dot plot shows caspase 3/7 (arbitrary units) activity in DMG cell lines normalised to 0 Gy DMSO control, when exposed to 24 Gy of radiation (RT) or Navitoclax (ABT-263 at 10 µM). Student t test, p-value shown, n = 3. (**F**) Quantification of Annexin V-expressing proliferating and senescent DMG cells following 6 and 12 h of Navitoclax or vehicle exposure. Data represent mean ± SD. Statistical significance was calculated using a two-tailed student’s t test.

Dose response curves for D + Q, Piperlongumine and Alvespimycin did not reveal the selective killing of senescent relative to proliferative DMG cells in any of the four cell lines tested (HSJD-DIPG14A, HSJD-DIPG007, SU-DIPG-IV and ICR-B117) (Supplementary Fig. 5). In contrast, Navitoclax showed a potent and selective efficacy on HSJD-DIPG14A, HSJD-DIPG007 and SU-DIPG-IV senescent cells, relative to proliferative cells (IC50 senescent cells: 0.01 µM, 0.04 µM, and 0.07 µM, respectively; IC50 proliferative cells: 2.37 µM, 2.55 µM, and 0.9 µM, respectively (Fig. 3A**; **Table 1**)**. The cell line ICR-B117 showed lower sensitivity to Navitoclax, relative to the other DMG cell lines (IC50 senescent cells: 0.34 µM; IC50 proliferative cells: 0.38 µM.

**Table 1:** IC50 values from proliferative and senescent H3K27M-altered human DMG cell lines cultured in the presence of anti-apoptotic protein inhibitors.

		IC50 (μM)		
Drug	Cell lines	Proliferative cells	Senescent cells	Senolytic index*
Navitoclax	HSJD-DIPG007	2.55	0.04	63.7
Venetoclax		26.26	1.87	11.9
Obatoclax		0.30	0.36	0.5
PZ18753B		7.81	0.02	390.5
DT2216		26.19	0.10	261.9
Nav-gal		3.74	0.10	37.4
Navitoclax	HSJD-DIPG14A	2.37	0.01	237
Venetoclax		3.62	2.66	1.3
Obatoclax		1.89	1.07	1.7
PZ18753B		18.18	0.02	909
DT2216		0.56	0.09	6.2
Nav-gal		1.31	0.15	8.7
Navitoclax	SU-DIPG-IV	0.90	0.07	12.8
Venetoclax		3.85	4.00	0.9
Obatoclax		1.04	0.39	2.6
PZ18753B		4.81	0.01	481
DT2216		0.33	0.01	33
Nav-gal		39.62	0.15	246.1
Navitoclax	ICR-B117	0.38	0.34	1.1
Venetoclax		3.18	4.40	0.7
Obatoclax		0.25	0.27	0.9
PZ18753B		0.63	0.14	4.5
DT2216		0.87	0.06	14.5
Nav-gal		1.66	0.14	11.8

* Senolytic index: IC50 proliferative cells/IC50 senescent cells.

Navitoclax can inhibit Bcl-2, Bcl-xL, and Bcl-w^37^. In order to elucidate the specific protein responsible for the senolytic effect of Navitoclax, we used several chemical BH3 mimetics with specific inhibitory profiles (Supplementary Table 2**)**. Drug sensitivity scores (DSS) were determined from dose–response curves data obtained in irradiated and non-irradiated cells. Our findings revealed low DSS values (DSS Range: 5.8–12.5) for Venetoclax, a Bcl-2 inhibitor, A-1210477, an MCL-1 inhibitor, and Obatoclax, a pan-Bcl2 inhibitor (Supplementary Fig. 6A, B and D; Fig. 3B). These low DSS values indicate low susceptibility for these inhibitors in our experimental setup. On the other hand, DSS values obtained for all inhibitors with strong affinity to Bcl-xL (Navitoclax, A-1331852, A-1155463) indicated higher sensitivity for all cell lines in the senescent state (DSS Range: 9.9–41) (Fig. 3B; Supplementary Fig. 6C and E). Furthermore, the differential DSS (dDSS), obtained from subtracting the DSS of non-irradiated (proliferative cells) from the DSS of radiation-induced senescent cells (RIS-DSS), were positive for all cell lines treated with Bcl-xL inhibitors, indicating that senescent DMG cells exhibited a preferential susceptibility to Bcl-xL inhibition rather than to Bcl-2 or MCL-1 inhibition (Fig. 3C).

Next, we compared the IC50 values obtained in DMG cell lines in this study with those from the Genomics of Drug Sensitivity in Cancer (GDSC) study for three different BH3 mimetics, Navitoclax, Venetoclax and Obatoclax (Fig. 3D). As the DMG cell lines used in our study are not represented in the GDSC database, we estimated the sensitivity of our DMG cell lines against the 967 cell lines of the GDSC^38^. The IC50s of Navitoclax in irradiated DMG cells were lower than 0.34 µM, ranking among the top 16% of most sensitive cell lines. Specifically, HSJD-DIPG-14A, HSJD-DIPG007, SU-DIPG-IV and ICR-B117 ranked 58^th^, 76^th^, 107^th^ and 157^th^ out of the 967 cell lines of reference **(**Fig. 3D). In contrast, the IC50s of Venetoclax were higher in senescent DMG cells (1.87–4.4 µM), and the 4 DMG cell lines ranked 119^th^ (HSJD-DIPG007), 154^th^ (HSJD-DIPG-14A), 194^th^ (SU-DIPG-IV) and 205^th^ (ICR-B117), clearly demonstrating a lower sensitivity compared with Navitoclax (Fig. 3D**, **Table 1). Senescent DMG cells were also less sensitive to obatoclax (ICR-B117: 394^th^; HSJD-DIPG-007: 441^st^; SU-DIPG-IV: 453^th^; HSJD-DIPG-14A: 628^th^) with IC50 of 0.27–1.07 µM (Fig. 3D**, **Table 1).

Finally, we sought to ascertain whether Navitoclax ablates the senescent DMG cells by apoptosis. Senescent (irradiated) and proliferative (non-irradiated) DMG cells were treated with 0.1 µM of Navitoclax or vehicle and caspase 3/7 activity quantified using either a luminescent assay or an automated real time measurement of Annexin V staining in a live-cell analysis system. Treatment with Navitoclax resulted in increased Annexin V signal and caspase activity, suggesting induction of apoptosis (Fig. 3E, F**)** Together, these analyses demonstrate that senescent H3K27M-altered human DMG cells are particularly sensitive to apoptosis through Bcl-xL inhibition.

### PROTAC-mediated Bcl-xL degradation and galacto-conjugated Navitoclax act as senolytic agents in senescent H3K27M-altered human DMG cells

A potential limitation of using Navitoclax clinically is the on-target toxicity affecting platelets, which leads to significant thrombocytopenia^39^. However, there have been two novel strategies to circumvent possible toxicity through the use of PROTAC-mediated Bcl-xL degradation^40^ and a galacto-conjugated form of Navitoclax that is specifically activated in senescent cells (Nav-Gal)^20^. Therefore, we sought to test the efficacy of Nav-Gal and two different senolytic PROTACs, named DT2216, targeting only Bcl-xL^40^ and PZ18753B, targeting both Bcl-xL and Bcl-2^41^.

**Fig. 4:**
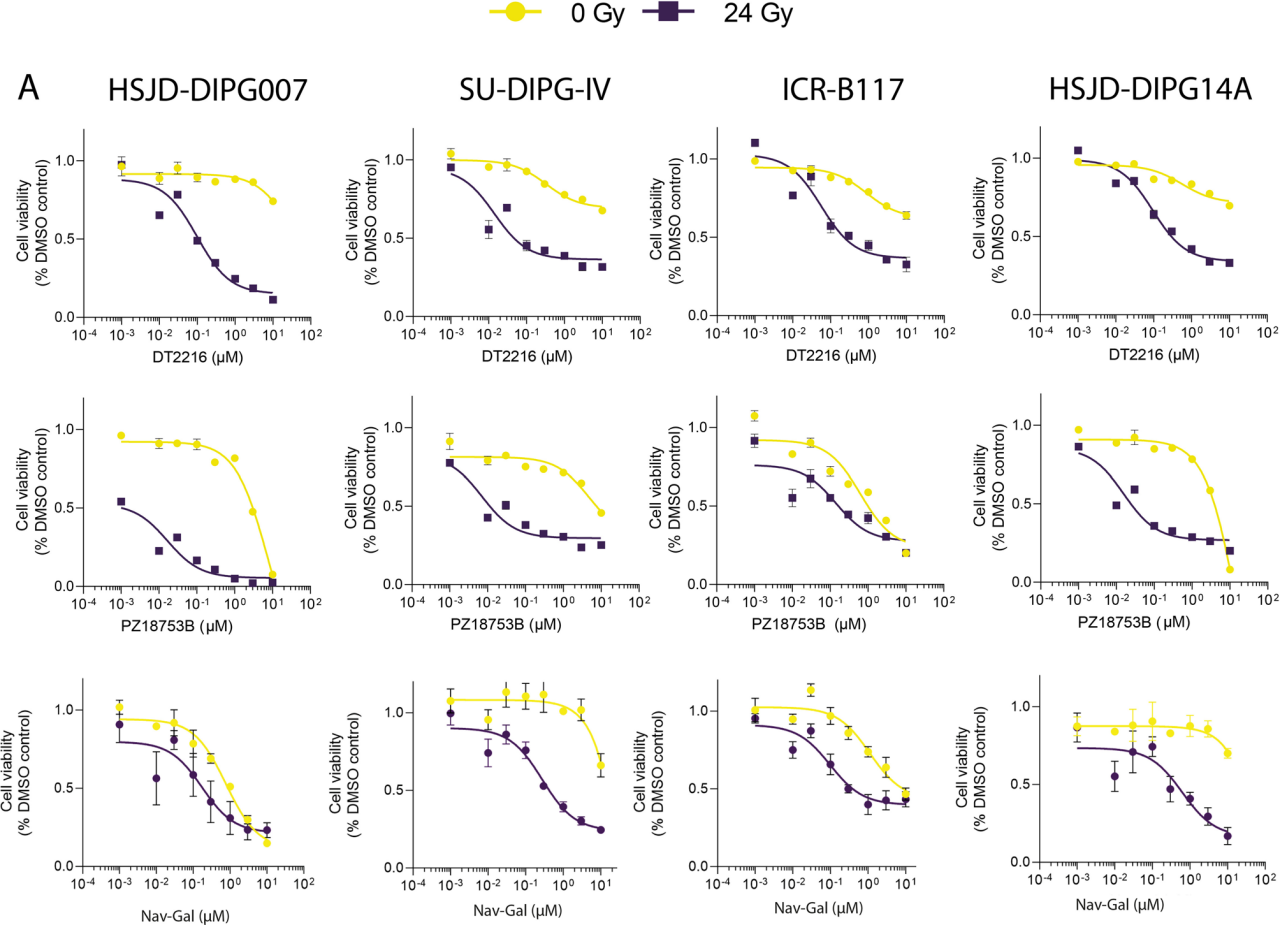
The prodrug Nav-Gal and Bcl-xL targeting PROTACs show efficient senolytic activity on senescent DMG cells. (**A**) Dose–response curves for the PROTACs DT2216 (targeting Bcl-xL) and PZ18753B (targeting Bcl-xL and Bcl-2), and the prodrug Nav-Gal on proliferating (yellow) and irradiated, senescent (purple) human DMG cells. Data is shown as mean ± SD.

To assess the senolytic capability of these compounds, dose response data were used to calculate the IC50s on senescent and proliferative cells (Fig. 4**; **Table 1). To compare these IC50s in a meaningful way, we decided to calculate the senolytic index (SI), defined as the ratio between the IC50s of proliferative and senescent cells. The SIs ranged from 8.54–264.66 (Nav-Gal), 45.93–266.16 (DT2216), 4.48–1163.89 (PZ18753B) and 1.13–165.78 (Navitoclax), suggesting that Nav-Gal and PROTACs exhibit a greater selectivity for targeting senescent over proliferative cells.

### Strong synergistic effect of a combination therapy of irradiation with Navitoclax

As the standard of care for treating DMG patients is RT, we aimed to assess the potential synergistic effect of combining radiation, which targets proliferative cells to induce cell death and senescence, with BH3-mimetics, which can ablate senescent cells. Cell viability was measured in HSJD-DIPG-14A, HSJD-DIPG-007, SU-DIPG-IV and ICR-B117, 72 h after combination of BH3-mimetic (Navitoclax, Obatoclax and Venetoclax) and increasing doses of radiation (0, 6, 12, 24, 36 Gy) (Supplementary Fig. 7A). Combinatorial effects were calculated using the Bliss independence model since the treatments combined were assumed to act independently using the Bliss independence model via SynergyFinder, the reported synergy scores represent the most synergistic values across the matrix. In this model, scores ranging from -10 to + 10 indicate an additive effect, whilst scores higher than + 10 demonstrate synergy^42^**.** Overall, the most synergistic (> 10) and additive (0–10) Bliss scores were obtained with a combination of RT with Navitoclax: HSJD-DIPG-14A, 22.1; HSJD-DIPG-007, 7.5; SU-DIPG-IV, 8.9; ICR-B117, 11.4 (Supplementary Fig. 7B). The combination of RT with Obatoclax was the least effective and not synergistic (HSJD-DIPG-14A, 6.3; HSJD-DIPG-007, 1.1; SU-DIPG-IV, 1.8; ICR-B117, 2.1) (Supplementary Fig. 7B). RT and Venetoclax combination led to a mixed response at inhibiting cell viability, and not synergistic HSJD-DIPG-14A, 18.3; HSJD-DIPG-007, 3.4; SU-DIPG-IV, 2.4; ICR-B117, 8.1 (Supplementary Fig. 7B). Together with the data shown previously, these results confirm that H3K27M-altered human DMG cells show a strong sensitivity to a combination therapy of RT and Bcl-xL inhibition.

### Combination therapy with radiotherapy and Navitoclax induces cancer cell death in a DMG PDX model

We next sought to assess in vivo the efficacy of a combination therapy of Navitoclax and RT against DMG. HSJD-DIPG-14A, SU-DIPG-IV and ICR-B117 cells have previously shown very low engraftment potential, and although HSJD-DIPG-007 cells engrafted with high efficiency in our hands, tumours showed little response to RT (Supplementary Fig. 8) possibly due to the dose regime used in this study, which could not increase above 12 Gy due to unacceptable toxicity in the NSG mice). The cell line SU-DIPG-VI has previously been shown to develop DMG tumours in the pontine area upon transplantation^43^, hence we decided to use this cell line. We first confirmed that SU-DIPG-VI cells also show a senescent phenotype in response to irradiation and sensitivity to Navitoclax in the senescent state (IC50, 0.27 mM; Senolytic index, 6.3) (Supplementary Fig. 9). In vivo pre-clinical data in aged non-human primates, has confirmed that navitoclax crosses the BBB and reaches concentration in the CSF able to ablate senescence and SASP responses^44^.

**Fig. 5:**
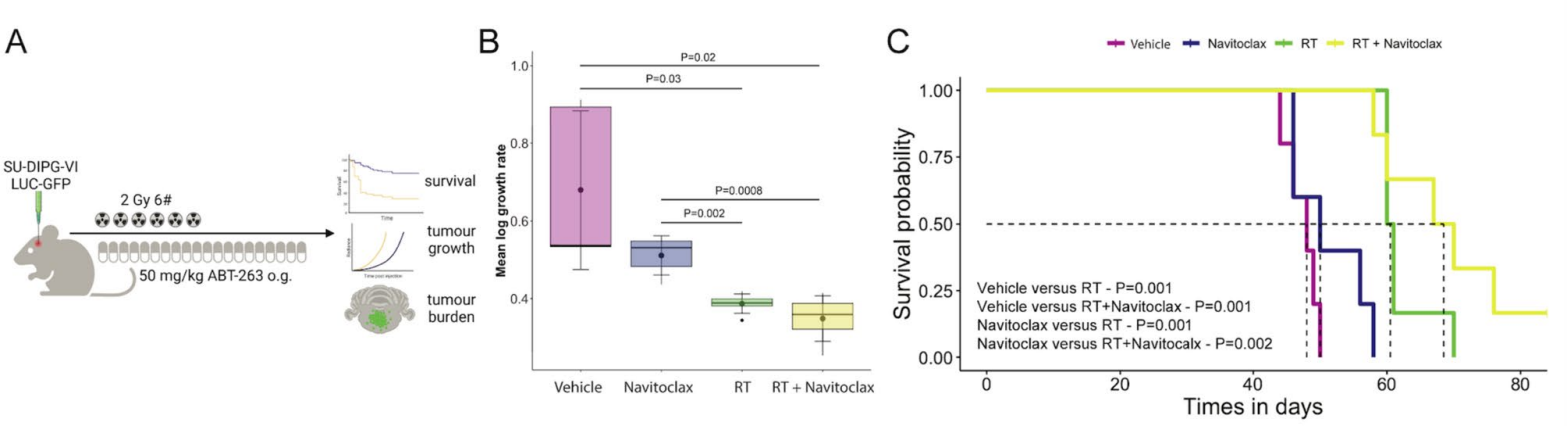
Combined Navitoclax and radiotherapy enhances anti-tumour response in vivo. (**A**) Schematic representation of the in vivo experimental design. Following orthotopic transplantation in NSG mice, tumour-bearing animals were randomized into four treatment groups: vehicle, Navitoclax, radiotherapy (RT), and RT plus Navitoclax. (**B**) Tumour growth dynamics assessed by relative luciferase radiance, expressed as Δlog radiance/time, showing a marked reduction in tumour growth in the RT and combination therapy groups compared with controls. (**C**) Kaplan–Meier survival curves illustrating improved survival in RT- and RT plus Navitoclax–treated mice, with the combination treatment showing a trend towards enhanced efficacy compared with RT alone that did not reach significancy.

To enable the identification of tumours in vivo, we generated eGFP-LUCF-SU-DIPG-VI cells, which express eGFP and luciferase, and used these cells in orthotopic transplantations in NSG mice (Supplementary Methods). A schematic of the preclinical experimental design is shown in Fig. 5A. After cell transplantation into the pons, BLI was used as an estimate of tumour burden, and tumour-bearing mice distributed into 4 groups of similar BLI levels, 20 days after transplantation (Supplementary Fig. 10). The experimental groups were as follows: (a) an untreated control group, which was not irradiated and received the vehicle used to prepare Navitoclax; (b) a Navitoclax-only group, which was not irradiated and received Navitoclax (50 mg/kg/day) via oral gavage, five days per week for four weeks; (c) a radiotherapy (RT)-only group, which received six fractions of 2 Gy/day (total 12 Gy, as higher doses resulted in unacceptable toxicity) together with the vehicle used to prepare Navitoclax; and (d) a combination therapy group, which received both RT and Navitoclax as described for groups (b) and (c).

Analyses of the tumour growth rates (TGR, defined as BLI change over time) showed no significant differences between the Navitoclax-only treated and untreated control vehicle groups (groups a and b) (Fig. 5B**)**. RT alone (group c) reduced TGR significantly in comparison with both, untreated vehicle control (group a, p = 0.03) and Navitoclax only group (group b, p = 0.002) (Fig. 5B**)**. Likewise, the combination of RT plus Navitoclax resulted in a significant reduction of TGR in comparison with groups a and b (p = 0.02 and p = 0.0008, respectively), and led to a trend towards a reduction that did not reach significance against the RT-alone group c (Fig. 5B).

Kaplan–Meier survival curves using log-rank tests were generated to analyse the survival probability of the four groups of mice (Fig. 5C). As with TGRs, significant differences in median survival were observed only when the RT-only (group c, 60.5 days) and the RT plus Navitoclax (group d, 68.5 days) groups were compared with the vehicle (group a, 48 days) and Navitoclax-only (group b, 50 days) groups (RT-only: p = 0.001 and p = 0.001; RT plus Navitoclax: p = 0.001 and p = 0.002). Although, the median survival was increased in the RT plus Navitoclax group (68.5 days) relative to the RT-only group (60.5 days), these differences did not reach significance (p = 0.18). Together, these data suggest that in this specific experimental paradigm, combination of RT + Navitoclax did not show a significant improvement over radiotherapy (RT) alone.

These in vivo results contrasted sharply with the strong senolytic effects of Navitoclax observed in RT-induced senescent DMG cells in vitro. To address this discrepancy, we conducted a new experiment to determine whether irradiation could induce senescence and thereby create a vulnerability to Navitoclax in DMG tumours in vivo. As before, eGFP-LUCF-SU-DIPG-VI cells were orthotopically transplanted into the pons. Tumour growth was monitored by bioluminescence imaging (BLI) at 20 days post-transplantation, after which mice were divided into four treatment groups: (a) unirradiated control group; (b) Navitoclax-only group, which was not irradiated and received Navitoclax (50 mg/kg/day) via oral gavage for 10 consecutive days; (c) radiotherapy (RT)-only group, which received six fractions of 2 Gy/day (12 Gy total); and (d) combination therapy group, which was treated with both RT and Navitoclax as described for groups (b) and (c).

**Fig. 6:**
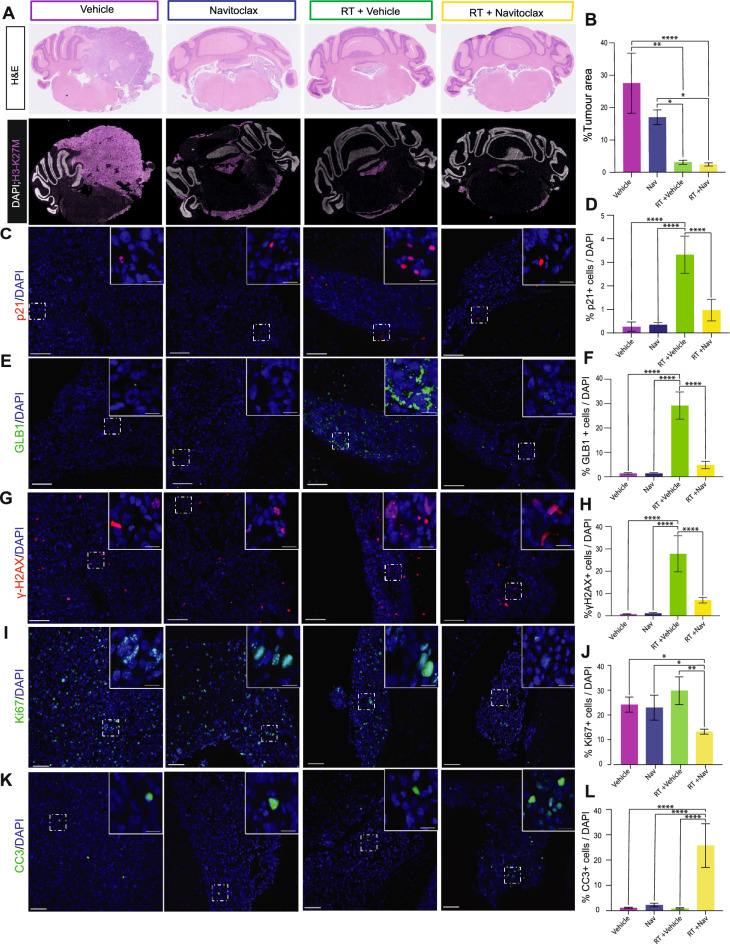
Radiotherapy induces senescence in vivo, while combination with Navitoclax enhances apoptosis and reduces proliferation. (**A**) Representative histological images of brain sections from each experimental group stained for H3-K27M. (**B**) Quantification of the tumour area occupied by H3-K27M–positive cells from (A). (**C–H**) Immunofluorescence staining of SU-DIPG-VI tumour sections for senescence markers p21^Cip^^1^ (C), GLB1 (E), and γ-H2AX (G), with quantification shown in (D, F, H). (**I–L**) Immunofluorescence staining for proliferation (Ki67; I) and apoptosis (cleaved caspase-3, CC3; K), with quantification shown in (J, L). RT induces a strong senescence response, while the combination of RT plus Navitoclax significantly reduces senescent cell numbers, increases apoptosis, and decreases proliferation 24 h after treatment. Scale bars: 100 μm. Data are mean ± s.e.m. (one-way ANOVA; *P* values shown; n = 3).

We assessed the tumours in the four experimental groups 10 days post-irradiation histologically and by immunofluorescence staining to assess the consequences of the treatments at the histological and cellular levels. Quantification of the tumour area showed no significant differences between untreated vehicle control and Navitoclax-only treated groups (groups a and b) (Fig. 6A, B). In contrast, treatment with RT alone or RT plus Navitoclax resulted in significant decreases in tumour area relative to groups a and b (RT-only: p = 0.0002 and p = 0.03; RT plus Navitoclax: p = 0.0001 and p = 0.02). No significant difference was observed between the RT and the RT plus Navitoclax groups (Fig. 6A, B).

To assess whether RT induced a senescent response in this experimental setting, we performed immunostaining for key senescence markers, including CDKN1A (p21^Cip^^1^), GLB1 (encoding the lysosomal β-galactosidase underlying SA-β-Gal activity), and γ-H2AX. Quantitative analysis showed a significant increase in the number of marker-positive cells in the RT-only group compared with both the vehicle control (group a; p = 0.0001) and the Navitoclax-only group (group b; p = 0.0001). Importantly, expression of all three markers was markedly reduced in the RT plus Navitoclax group relative to RT alone (p = 0.0001) (Fig. 6C–H**).** These results mirror our in vitro findings, suggesting that RT induces senescence in DMG cells and that Navitoclax eliminates the RT-induced senescent population.

To further investigate this finding, we performed immunostaining for cleaved caspase-3 (CC3) as a marker of apoptosis and Ki67 to assess cell proliferation (Fig. 6I,K). Ki67 expression did not differ significantly among the vehicle control, Navitoclax-only, and RT-only groups (groups a, b, and c). In contrast, the RT plus Navitoclax group exhibited a significant reduction in the Ki67 proliferative index compared with RT alone (p = 0.00023) (Fig. 6I,J). Importantly, CC3-positive cells were nearly absent in the vehicle, Navitoclax-only, and RT-only groups (0.9–2.3%). However, the RT plus Navitoclax combination resulted in a marked increase in apoptosis, with 25.7% CC3-positive cells—a significant elevation relative to RT alone (p = 0.00001) (Fig. 6K,L). Taken together, these analyses support the conclusion that RT induces senescence in DMG cells in vivo and creates a corresponding vulnerability to Navitoclax. Nonetheless, within the constraints of this experimental setting, these effects are not sufficient to produce measurable differences in tumour growth or mouse survival.

## Discussion

In this study, we have demonstrated that ionising radiation results in both senescence induction (Figs. 1 and 2) and increased sensitivity to BH3-mimetics (Fig. 3) in a set of heterogeneous H3K27M-altered human DMG cells. Using specific inhibitors, we show that the survival of senescent DMG cells depends mostly on the anti-apoptotic protein Bcl-xL rather than the family members Bcl-2 or Mcl-1 (Figs. 3B-D and 4). We reveal that irradiation act synergistically with Navitoclax to induce the cell death of DMG cells in the senescent state (Fig. 3E, F**; **Supplementary Fig. 7). However, not all the DMG cell lines tested exhibit the same sensitivity to Navitoclax, and in particular ICR-B117 and SU-DIPG-VI cells are less sensitive (IC50 0.34 μM and 0.27 μM, respectively), while HSJD-DIPG-14A, HSJD-DIPG-007 and SU-DIPG-IV are more sensitive with IC50 values of less than 70 nM (Table 1).

Senescence induction and sensitivity to Navitoclax is supported by the data obtained in a PDX model of DMG, despite using a cell line (SU-DIPG-VI) with less sensitivity in vitro. A fractionation regimen of 2 Gy per day for 6 days leads to significant tumour reduction in vivo, increased mouse survival and long-term accumulation of senescent cells. Moreover, combination of irradiation with Navitoclax results in significant reduction in the expression of senescent markers as well as Ki67-positive cells, and the concomitant increase of cleaved caspase 3 expression, indicating cell death (Fig. 6**; **Supplementary Fig. 9). These findings are supportive of senescence induction by RT, and senescent cell ablation caused by Navitoclax. Further molecular characterization, e.g. single cell RNA sequencing will be required to characterize further this senescence response in different DMG mouse models.

Although our in vitro studies demonstrate a marked synergy between irradiation and Navitoclax, the in vivo paradigm used did not detect significant differences in tumour growth rate or survival between the groups treated with only irradiation and the irradiation plus Navitoclax. We observed only a non-significant trend towards reduced tumour growth rate and increased mouse survival in the combination group compared with the irradiation-only group (Fig. 5B-C). It is possible that further refinement of the experimental setting may be required, e.g., longer treatment with a higher dose of Navitoclax, and/or higher irradiation dose), but these alterations may cause further toxicity. For example, we were not allowed to irradiate the mice with a higher dose than 12 Gy (2 Gy for 6 days) to avoid unacceptable toxicity, which is much less than the standard treatment in patients (54 Gy in 30 fractions of 1.8 Gy). Likewise, long Navitoclax treatment is associated with toxicity in mice. Further, it was noted that there was leptomeningeal disease in some mice, which may further affect drug-distribution and tumour microenvironment (Fig. 6). Nonetheless, our data provide proof-of-principle irradiation increases the expression of senescent markers in DMG tumours in vitro and in vivo, and Navitoiclax can ablate these cancer cells.

Previous studies have shown that DMG cells can be induced into senescence by inhibition of BMI1 and EZH2^45–47^. Moreover, senescent cells induced through BMI1 inhibition are vulnerable to BH3-mimetics both in vivo and *in vitro*^46^, indicating the senescent response could be exploited therapeutically. Together with our data these findings hold high translational interest as compounds inhibiting Bcl2 family proteins, including Bcl-xL, such as Navitoclax, PROTAC, and Nav-Gal, are already in advanced clinical development, making them potential candidates for clinical phase I/II trials in paediatric DMG.

The sensitivity of senescent cells to Bcl-xL inhibition has also been shown in other brain tumours. The high-grade adult glioma, glioblastoma (GBM) is known for its aggressive and invasive nature, making it one of the most challenging brain tumours to treat. GBM can undergo senescence following radiation or temozolomide treatment and preclinical studies have shown that targeting senescent cells through Bcl-xL inhibition could be a valuable therapeutic strategy for improving GBM treatment outcomes^2,5,23,48^. Likewise, sensitivity to Bcl-xL inhibition has been observed in cell lines of pilocytic astrocytoma, the most common brain tumour in children, and adamantinomatous craniopharyngioma, the most common childhood pituitary tumour, suggesting a potential therapy against these tumours^27,49^.

Recently, Venetoclax, also known as ABT-199, a Bcl-2 inhibitor, has been shown to cooperate with irradiation in controlling paediatric DMG^50^. Notably, in our study, the role of Venetoclax has not been found to be critical in maintaining survival of senescent, irradiated cells. Instead, we show that Bcl-xL inhibition is a more crucial target for senolytic activity (Table 1).

Other BH3 mimetic compounds, including novel PROTACs and Nav-Gal confirmed the marked sensitivity of senescent DMG cancer cells to BCL-xL inhibition (Table 1**; **Fig. 4). Future work including siRNA for Bcl knockdown could exemplify this dependency further. Of note, PROTAC PZ18753B, which targets for degradation both anti-apoptotic proteins Bcl-xL and Bcl-2, results in the lowest IC50 values and highest senolytic scores in our study (Table 1), suggesting potential functional redundancy. In summary, we show the efficacy of BH3 mimetics as senolytic compounds in senescent DMG cell lines, both in vivo and in vitro. The ongoing clinical development of less toxic and more selective Bcl-xL inhibitors and their evaluation in clinical trials for other tumours present exciting prospects for future therapeutic interventions in brain tumours, including DMG.

## Materials and methods

### Gamma-ray irradiation of DMG cells

H3K27M-altered human DMG cells (Supplementary Table 1; Supplementary Methods) were grown under adherent stem cell conditions as described^51^. Adherent cultures were used as opposed to neurospheres to improves consistency for imaging and drug screening without significantly altering key phenotypic markers. ^137^Cs γ-ray irradiation at a dose rate of 1.76 Gy/min was conducted using an IBL 437C (CIS Bio-International, Codolet, France). DMG cells were cultured to less than passage 10 prior to induction of senescence through irradiation. Cells were dissociated using accutase (Sigma), counted using Trypan blue and cells resuspended in 1 ml of TSM-C and irradiated in 15 ml falcon tubes. Cells were plated after irradiation (day 0) and left for 5 days before assessing senescent and SASP responses (Day 5), when molecular (RNA sequencing, ELISA) and immunocytochemical analyses were performed. Media was changed at Day 1 and Day 3, as cell debris due to cell death was abundant around days 2 and 3. To avoid false-positivity due to differences in confluency, irradiated (senescent) and proliferative cells were collected for molecular or cellular analyses at 70–80% confluency.

### Senescence-associated beta-galactosidase, EdU and apoptosis staining

Senescence and EdU staining were performed as described^26^. A minimum of 400 cells were counted per condition. Caspase 3/7 activity assay and Annexin V staining were used to quantify cell apoptosis. Full details can be found in Supplementary Methods.

### Dose response cell assays

Upon irradiation, cells were plated at a density of 5,000–10,000 cells/well on laminin-coated 96-well plates (black opaque) in a minimum of triplicates and allowed to sit for 18 h. Medium was replaced the next day and five days post-irradiation, fresh media supplemented with drugs were added to each well and incubated at 37 °C in 5% CO2, 95% humidity for 3 days (Supplementary Table 2; Supplementary Methods). Cell viability was assessed by the CellTiter-Glo luminescent cell viability assay (Promega). SynergyFinder (https://synergyfinder.fimm.fi) was used for interactive analysis and visualization of drug combination profiling data following the Bliss independence model^52^. To calculate drug sensitivity scores (DSS), a publicly available online platform was used (Breeze version 2.0; https://breeze.fimm.fi/94912_mc42ntaxmtywmcaxnzq0mdk4njix/index.php)^53,54^.

### Immunofluorescence staining

Immunofluorescence staining was carried in 96 well plate at desired timepoints (usually 5 days following radiation unless otherwise stated) using previously described protocols^26,27^. Full details in Supplementary Methods and Supplementary Tables 3 and 4.

### RNA sequencing and bioinformatic analysis

RNA was extracted from both non-irradiated (proliferative) and irradiated (senescent; five days post-irradiation) human DMG cells using the Rneasy Micro kit (Qiagen). Total RNA integrity was validated using Agilent’s 4200 Tapestation, with all samples having RIN values > 9.4. cDNA library preparation used 250 ng of total RNA and the KAPA mRNA HyperPrep Kit. High-quality libraries were confirmed on the Agilent TapeStation 4200. Samples from the three DMG cell lines were normalized to 4 nM using the Normalase assay, and independently sequenced on the NextSeq 500 instrument with a 75 bp pair-end run. Fastq files were aligned to the human genome UCSC hg38 using RNA-STAR 2.5.2b and differential expression (DE) analysis performed with DESeq2^55^. Gene ontology analysis was performed using the GOSeq package in R^56^. Gene Set Enrichment Analysis GSEA using version 4.2.3 with gene sets from MSigDB^57,58^.

### Meso Scale Discovery (MSD) for cytokine and chemokine detection in cell culture supernatant

Collected supernatants from irradiated cells were centrifuged and stored at − 80 °C. To avoid contamination of cytosolic components of dying cells after irradiation, media was changed on one and three days after irradiation. Cytokine levels were measured in conditioned medium at day 5 post-irradiation using a Meso Scale Discovery multiplex kit (Meso Scale Diagnostics, Rockville, MD), according to the manufacturer’s instructions. IFN-γ, IL-1β, IL-2, IL-4, IL-6, IL-8, IL-10, IL-12p70, IL-13 and TNF-α were measured by V-PLEX MSD assay, and MIG (CXCL9) and IL-18 were measured by U-PLEX MSD assay. The plates were analyzed on the MSD instrument (QuickPlex SQ120). Data were log transformed for visual representation.

### Generation of PDX mice, brain irradiation and tumour growth visualisation

A total of 250,000 eGFP-LUCF-SU-DIPG-VI cells in 2–3 μL were stereotactically implanted in the pontine area of NOD-SCID male mice (Charles River) using a 26SG Hamilton syringe using a rate of ~ 1 μL/min. Co-ordinates used were 1.0 mm lateral to midline, 0.8 mm posterior to lambda, and –4.2 mm deep to cranial surface. A small pocket was created for the cells, which were injected -3.2 mm deep to the cranial surface. At the completion of infusion, the syringe needle was allowed to remain in place for a minimum of 2 min, then slowly manually withdrawn to minimize backflow of the injected cell suspension. The skin was closed with VetBond™ Adhesive. Mice were weighed daily for one week, and dosed for 48 h with Metacam, at 5 mg/kg. Whole brain irradiation was carried out using a Small Animal Radiation Research Platform (SARRP, Xstrahl Inc.). Mice received a total of 12 Gy in 2 Gy fractions (six consecutive days), delivered as an arc, targeted to maximise delivery to the whole brain (the beam was directed to avoid the mouth and jaw area to reduce mucositis). ABT-263 was administered to mice by gavage at 50 mg per kg body weight per day (mg/kg/d). Tumour growth was monitored using an IVIS Spectrum in vivo imaging system (IVIS Living Image software version 4.8.2, IVIS® Lumina Series III (Perkin-Elmer, Beaconsfield, UK). Ten minutes following subcutaneous injection of D-luciferin (Perkin Elmer), bioluminescent images were acquired under isoflurane anaesthesia. Tumour size was quantified by calculating total flux (photons/sec) using IVIS software. Tumour growth rate was quantified using total flux/time, using a linear mixed-effects model. Defined end points were used to avoid suffering and mice showing moderate symptoms and deemed to be irrecoverable were humanely culled before these adverse effects progressed in severity. The mouse experiments were approved by a UCL ethical review panel and covered by a Project Licence granted to the corresponding author by the UK Home Office. All methods used on mice were in accordance with standards set by the UCL Biological Services Unit, Home Office and the ARRIVE guidelines.

## Supplementary Information

Supplementary Information 1.

Supplementary Information 2.

## Data Availability

The RNA sequencing data have been deposited at Gene Expression Omnibus (GEO Submission GSE291046).
