# Supplementary material for: Navitoclax acts synergistically with irradiation to induce apoptosis in preclinical models of H3K27M-altered diffuse midline glioma
Source: Sci Rep. 2025 Dec 22;15:45094. doi: 10.1038/s41598-025-32676-6 (PMC12749289; doi:10.1038/s41598-025-32676-6)
Supplement: Supplementary file 1 — Supplementary Information 1. [file 41598_2025_32676_MOESM1_ESM.docx]

**Supplementary Methods**

**Primary DMG cell line culture**

H3K27M-altered human DMG cells (**Supplementary Table 1**) were grown under adherent stem cell conditions using flasks coated with laminin (10ug/ml) (R&D systems, Bio-techne) in tumour stem media complete (TSM-C). TSM base (TSM-B) was made using 250mL Neurobasal-A Medium (Invitrogen), 250mL DMEM/F-12 (Invitrogen), 1% HEPES buffer solution (ThermoFisher), Sodium pyruvate MEM 100mM (ThermoFisher), MEM non-essential amino acids solution 10mM (ThermoFisher), GlutaMAX-I supplement (ThermoFisher), Antibiotic-antimycotic (ThermoFisher). Prior to use TSM-B was supplemented with B-27 without vitamin A (Invitrogen) and the growth factors epidermal growth factor (EGF, 20 ng/ml Peprotech), basal fibroblast growth factor (bFGF, 20 ng/ml, Peprotech), platelet-derived growth factor (PDGF-AA and PDGF-BB, 10 ng/ml, Peprotech) and 0.2% heparin sulphate (Stemcell technologies), TSM-C was used within two weeks. All cell lines were routinely confirmed mycoplasma negative using the MycoAlert™ PLUS Mycoplasma Detection Kit (Lonza, catalogue no. LT07-710) and STR profiled.

**Senescence-associated beta-galactosidase staining**

Senescence was verified by staining for senescence-associated-β-galactosidase (SA-β-gal) using a commercially available SA-β-gal staining kit (Sigma, catalogue no. CS0030), following manufacture instructions. A minimum of 400 cells counted per condition. Positive (blue) cells are expressed as a percentage of total cell number.

**EdU staining**

EdU (10µmol final concentration) diluted in TSM-C was added to the cells overnight. Click-it EdU (ThermoFisher) reaction was carried out as per manufacturers protocol. Briefly, media was removed, cells were washed with 1X PBS, and fixed with 4% Paraformaldehyde for 15 minutes. Cells were then washed with 3% BSA and permeabilised with 0.5% Triton-X100. Cells were washed a twice with 3% BSA and Click-it EdU reaction cocktail (Alexa Flour 488, 597 were used) was added to the cells for 30 minutes. A further wash with 3% BSA and nuclei were counter stained with Hoescht for 30 minutes.

**Drugs for in vitro and in vivo testing**

Navitoclax (ABT-263, catalogue no. 11500, Cayman Chemical), ABT-737 (catalogue no. ab141336, Abcam), Dasatinib (catalogue no. S1021, Selleckchem), Quercetin (catalogue no. S2391, Selleckchem), Piperlongumine (catalogue no. CAY-11006, Selleckchem), Alvespimycin (catalogue no. hy-12024, Selleckchem), BCL specific agents A1155463 (catalogue no. S7800, Selleckchem), A1331852 (catalogue no. S7801, Selleckchem), A1210477 (catalogue no. S7790, Selleckchem), Obatoclax Mesylate (GX15-070) (catalogue no. S1057, Selleckchem). PROTACs DT2216 and PZ18753B were obtained from Daohong Zhou (University of Florida) under MTA (5934207). Nav-Gal was obtained from Ramón Martínez-Máñez (IDM, University of Valencia) (**Supplementary Table 2**). All drugs were dissolved in DMSO and stored in aliquots at −20°C. Drugs were diluted in cell culture medium and added to the cells for the durations and concentrations indicated. Navitoclax (ABT-263) (100 mg, ApexBio), was weighed and vehicle (ethanol:polyethylene glycol 400:Phosal 50 PG) was added, individual aliquots were prepared, prior to administration drug was sonicated on ice into solution using Diagenode Bioruptor®, high frequency, 30 second intervals, for 15 minutes. ABT-263 was administered to mice by gavage at 50 mg per kg body weight per day (mg/kg/d) for 5 days for two cycles.

**Assessment of apoptosis**

Caspase 3/7 activity assay was determined using 100μl Caspase-Glo® 3/7 Reagent (Promega), which was added directly to the cells in 96-well plates and incubated for 1 hour before recording luminescence on a Microplate luminometer - GloMax® Navigator - Promega. Each point represents the average of 3 wells. The "no cell" blank control value has been subtracted from each. Annexin V cell viability analysis used the Annexin V Fluorescent Reagent (Essen Bioscience), which was added to the media, together with vehicle (0.1% DMSO) or 0.1 μM Navitoclax for 48 hours. Images were collected every 2 h with an Incucyte® S3 Live-Cell Analysis System microscope (Essen Bioscience) over time. A total of 5 pictures per well were analysed using the IncuCyte ZOOM™ software analyser, and total and annexin V-positive cells were counted using ImageJ software.

**Immunofluorescence staining**

Cells were fixed for 15 minutes using 4% PFA (w/v, in PBS) followed by washing 3 times with PBS. Cells were then permeabilised using 0.2% Triton X-100 (v/v, PBS) for 10 min and then washed twice with PBS to halt permeabilisation. Non-specific antibody binding was blocked by incubation with a blocking solution for 1 hour at RT. Blocking solution contained 1% BSA (w/v, PBS) supplemented with 1X Fish Skin Gelatin (v/v, PBS). Primary antibodies (**Supplementary Table 3**) were diluted in blocking solution and cells incubated with primary antibody solution for 1 hour at RT. Following incubation, primary antibody was then removed by washing 3 times with PBS. Secondary antibodies (**Supplementary Table 4**) conjugated to Alexa-594 or Alexa-488 fluorophores were then diluted in blocking solution added to wells to be incubated in dark for 1 hour. Secondary antibody was then removed by washing 3 times with PBS and nuclei counterstaining with 1μg/mL DAPI (w/v, PBS) for 10 minutes. Cells were then washed with PBS three times. Immunofluorescence image acquisition was performed using an automated Opera Phenix high-throughput confocal microscope (Perkin Elmer). Wells were imaged using a 10x, 20x or 40x water objective binning of images were used to reduce image file sizes. Fluorophores were imaged using pre-set ‘DAPI’, ‘AlexaFluor 594’ & ‘AlexaFluor 488’ wavelengths on microscope respectively. Depending on staining at least 20 fields per well were captured for 10x, 20x and 40x objectives respectively. High content image analysis was carried out using the Opera Phenix Harmony software (Perkin Elmer). DAPI nuclear counterstain was used to segment cells.

**LysoTracker staining**

For live imaging, Lysosomes were visualised by incubation of 100 nM LysoTracker Deep Red (Molecular Probes, Thermo Fisher) for 30 minutes in complete media. Cells were counterstained with Hoescht for 10 minutes at a concentration of 1μg/ml, diluted in complete media. Fluorophores were imaged using pre-set ‘DAPI’, ‘AlexaFluor 647’ wavelengths on the microscope respectively. 10 fields per well were captured using a 40x water objective. High content image analysis was carried out using the Opera Phenix Harmony software (Perkin Elmer). Hoescht nuclear counterstain was used to segment cells. Cytoplasmic fluorescence intensity was measured per nuclei.

**Generation of eGFP-LUCF-SU-DIPG-VI cells**

For cell transduction, the plasmids pBM N(CMV-copGFP-Luc2-Puro), pMD2.G and psPAX2 were used (Addgene plasmids 80389, 12559 and 12260). Cells were seeded in 24 well plates, and transduced at 70% confluence. Medium was removed by aspiration and replaced with fresh complete medium containing 5-8 µg/ml polybrene (Millipore). Viral supernatant was added to achieve a multiplicity of infection (MOI) of 25%. After 18 hours, the medium was replaced with fresh complete medium to remove lentivirus and polybrene. Transduced cells were expanded and sorted using flow cytometry assisted cell sorting (FACS), based on GFP fluorescence sorted cells were expanded and stored in liquid nitrogen.

**Pontine cell injections**

A single-cell suspension (eGFP-LUCF-SU-DIPG-VI) was prepared immediately before implantation in 8-10 weeks old NOD-SCID male mice (Charles River). For intracranial procedures, animals were anesthetised with isoflurane. The cranium was exposed via midline incision under aseptic conditions and 1×1mm deep hole was made through the skull to the dura. Mice were placed in a stereotactic apparatus and 250,000 cells in 2-3 μL were stereotactically implanted in the pontine area using a 26SG Hamilton syringe using a rate of ~ 1 μL/min. Co-ordinates used were 1.0mm lateral to midline, 0.8mm posterior to lambda, and –4.2 mm deep to cranial surface. A small pocket was created for the cells, which were injected -3.2 mm deep to the cranial surface. At the completion of infusion, the syringe needle was allowed to remain in place for a minimum of 2 minutes, then slowly manually withdrawn to minimize backflow of the injected cell suspension. The skin was closed with VetBond™ Adhesive. Mice were weighed daily for one week, and dosed for 48 hours with Metacam, at 5 mg/kg.

**Mouse brain irradiation**

Irradiations utilised an Xstrahl Small Animal Radiation Research Platform (SARRP), (Xstrahl Inc., Suwanee, GA), with a Comet MXR-225/22 x-ray tube (0.8 mm integral Be filtration). The SARRP was used for cone beam CT imaging, at 60 kVp and 0.8 mA with 1 mm Al filtration; and for treatment, at 220 kV and 13 mA, with 0.15 mm Cu filtration (half value layer 0.847 mm Cu, reference dose rate at 2cm depth, approx. 3.1 Gy min^−1^. Mice were anaesthetised with isoflurane and positioned for optimal brain targeting on a 3D printed bed. Target position was identified from CT image reconstructed from 360 projections (1° increments/ projection, approximately 0.01 Gy total radiation dose), using an FDK algorithm, with voxel size from between 0.01 and 5 mm. Muriplan software was used to set tissue radiodensity thresholds, and enable treatment planning; setting a target iso-centre for the injection site of the brain, with dose calculation computed via superposition/convolution. Mice received a total of 12 Gy in 2 Gy fractions, delivered as a 190º arc, targeted to maximise delivery to the whole brain (excluding the olfactory bulbs). The beam was collimated, to match the treatment volume, with beam cross section 10 × 10 mm^2^. The time calculated to deliver 2 Gy to individual mice was approx. 51 sec, giving an indicated dose rate of 0.0395 Gy/sec.

**Supplementary Figures**

**Suppl. Figure 1**

**Supplementary Figure 1. Irradiation doses of 4 and 8 Gy induce SA-β-gal expression and reduce BrdU incorporation. (**A) Representative images showing SA-β-gal staining in unirradiated control and irradiated SU-DIPG-IV and HSJD-DIPG007 cells following 4 Gy and 8 Gy doses of radiation. (B) Representative images of EdU staining in unirradiated control and irradiated DMG cells. (C) Quantification of SA-β-gal–positive cells from experiments shown in (A). (D) Quantification of EdU-positive cells from experiments shown in (B). Data represent mean ± SD. Colours correspond to radiation dose as indicated in the legend. Statistical analysis was performed using one-way ANOVA. Scale bar: 50 μm.

**Suppl. Figure 2**

**Supplementary Figure 2. Fractionation leads to a senescent phenotype as single doses of radiation.** (A) Schema of experimental procedure for fractionation. DMG cancer cells received a total of 24 Gy, in 12 fractions of 2 Gy/day. Senescent was assessed 24 hours after the last irradiation dose. (B) Representative images of SA-β-gal and EdU staining of unirradiated and irradiated DMG cells. (C) Quantification of EdU and SA-β-gal positive cells following radiation treatment (D) qRT-PCR of irradiated cells following fractionation schedule, fold change relative to GAPDH shown as a heatmap (red – upregulated, blue downregulated, relative to control). One-way ANOVA. Scale bar = 50μ.

**Suppl. Figure 3**

**Supplementary Figure 3. Gene ontology and hallmark gene set enrichments analyses of irradiated and non-irradiated H3K27M-DMG cells.** (A) Immunofluorescence staining against p27 and p57 of DMG cells following RT (24 Gy). Scale bar = 100µ. (B) Quantification of A, a violin plot of fluorescence intensity (arbitrary units) demonstrated for CDKIs p21, p27 and p57. (C) Immunofluorescence staining against CRYAB of DMG cells following RT (24 Gy). Scale bar = 100µ. (D) Quantification of C, a violin plot of fluorescence intensity (arbitrary units) demonstrated for CRYAB. (E) Unsupervised hierarchical cluster heatmap showing top 200 differentially expressed genes between irradiated and proliferating DMG cells. Some top downregulated and upregulated genes’ symbols are annotated. (F) Volcano plot showing differentially expressed genes between irradiated and proliferating cells. Some top downregulated and upregulated genes’ symbols are annotated. There are more genes significantly upregulated in the senescent population than downregulated when compared with the non-senescent. Top upregulated genes such as MMP3, IL1B, CXCL8 are associated with the SASP (Log_2_ Fold-Change cut off at > 2 (yellow and purple dots), p-value shown is Log_10_ adjusted P-value, cut-off at P<0.01. (G) Heatmap showing a subset of differentially expressed genes representing the cell cycle KEGG gene set. (H) Heatmap showing a subset of differentially expressed genes representing the lysosomal compartment KEGG gene set. (I-J) Bar plot of Hallmark gene set enrichments (I) and depletions (J) following radiation. (K-L) Dot plots showing upregulated (K) and downregulated (L) gene ontology. p-value shown is adjusted p-value. Gene ratio = Genes expressed/Gene set total.

**Suppl. Figure 4**

**Supplementary Figure 4. Following irradiation HSJD-DIPG-14A cells exhibit a robust senescence phenotype, and are sensitive to Navitoclax.** (A) Cell confluency of HSJD-DIPG-14A cell line treated with single doses of irradiation (0, 12, 24, 36 Gy). Cells were monitored over a 35 day period. Error bar mean±SD. Colour relating to treatment. Legend colour representation of doses of radiation used. One-way ANOVA. (B) Representative images of SA-β-gal staining and EdU incorporation of unirradiated control and irradiated HSJD-DIPG-14A cells after different doses of radiation. SA-β-gal staining and EdU incorporation was assessed at day 5 post-irradiation. Scale bar = 50μ (C) Quantification of SA-β-gal positive and EdU positive cells out of total DAPI+ nuclei following radiation treatment. (D) Immunofluorescence staining for p21, pRB, γH2AX and LysoTracker live cell staining of HSJD-DIPG-14A cells following RT (24 Gy). Scale bar = 100µ. (E) Immunofluorescence staining for SASP factors CCL2, CX3CL1, IL1B, IL6 of DMG cell lines following RT (24 Gy).

**Suppl. Figure 5**

**Supplementary Figure 5.** **Senescent DMG cells do not respond to the senolytic agents Dasatinib plus Quecertin (D&Q), Piperlogumine or Alvespimycin**. Assessment of viability by Cell-Titer-Glo of irradiated senescent (purple) or unirradiated proliferating (yellow) DMG cells treated for 72 hours with Dasatinib and Quecertin (D&Q), Piperlongumine, Alvespimycin, at indicated concentrations. Shown are mean ± SD of three technical replicates.

**Suppl. Figure 6**

**Supplementary Figure 6. Senescent DMG cells are sensitive to the specific Bcl-xL inhibitors A1331852 and A1155463.** Assessment of viability by Cell-Titer-Glo of irradiated senescent (purple) or unirradiated proliferating (yellow) DMG cells treated for 72 hours with (A) Venetoclax, (B) Obatoclax, (C) A1331852, (D) A1210477, (E) A1155463 at indicated concentrations. Shown are mean ± SD of three technical replicates.

**Suppl. Figure 7**

**Supplementary Figure 7. Assays combining radiotherapy and three BH3 mimetics reveal Navitoclax as the most synergistic**. (A) DMG cells were treated with Navitoclax, Obatoclax and Venetoclax at 8 doses (0.001-10 µM) and radiotherapy at 0, 6, 12, 24, 36 Gy. Viability was assessed after three days of treatment using Cell-Titre Glo. Cell viability was depicted as a heatmap where yellow is 100% viability and purple is 0% viability (B). Bliss synergy scores were conducted by using synergy finder, using dose response curves.

**Suppl. Figure 8**

**Supplementary Figure 8. A fractionated dose of 12Gy fails to control tumour growth in DIPG007 transplanted NSG mice.** (A) Preclinical experimental design. Mice received a total of 12 Gy in 2 Gy fractions (six consecutive days, days 0-6 in graph), delivered as an arc, targeted to maximise delivery to the whole brain (the beam was directed to avoid the mouth and jaw area to reduce mucositis). ABT-263 or vehicle was administered to mice by gavage at 50 mg per kg body weight per day (mg/kg/d) for 5 days for two cycles (10 days total). (B) Growth rate and Bioluminescence (radiance, photons/sec) measured over course of experiment. (C) Radiance over time graphs of mice treated with RT plus vehicle (yellow) or RT and Navitoclax (purple). Note that RT treatment days 0-6 did not reduce BLI in either of the groups.

**Suppl. Figure 9**

**Supplementary Figure 9.** **Following radiation SU-DIPG-VI cells exhibit a senescence phenotype and are sensitive to Navitoclax.** SU-DIPG-VI cells were irradiated with single doses of radiation (0, 12, 24, 36 Gy) and assayed 5 days later. (A) Representative images of SA-β-gal staining of unirradiated proliferative control and irradiated senescent DMG cells after 24 Gy radiation. (B) Representative images of EdU staining of unirradiated proliferative control and irradiated senescent DMG cells after 24 Gy radiation. (C, D) Quantification of SA-β-gal positive cells (C) and EdU positive cells (D) following radiation treatment. (E) Assessment of cell viability by Cell-Titer-Glo of irradiated (purple) or proliferating (yellow) SU-DIPG-VI DMG cells, treated for 72 hours with Navitoclax at indicated concentrations. Shown are mean ± SD of three technical replicates. One-way ANOVA.

**Suppl. Figure 10**

**Supplementary Figure 10. BLI images of mice treated with either vehicle, Navitoclax, RT-only and RT plus Navitoclax.** Representative *in vivo* bioluminescent images of luciferase-expressing DMG tumour cells before, during and after treatment. The scale bar adjacent to the image displays bioluminescence counts (photons/second/cm^2^).

**Supplementary Table 1. Features of the K27M-altered DMG cell lines used in this study.**

| **Cell line ID** | **Clinical diagnosis** | **Location** | **Age (yrs)** | **Sex** | **Histology** | **WHO** | **Source** | **Survival (months)** | **Outcome** | **p.K27M mutated gene** | ***TP53*** | ***ACVR1*** |
| --- | --- | --- | --- | --- | --- | --- | --- | --- | --- | --- | --- | --- |
| **HSJD-DIPG-007^1*^** | DIPG | Pons | 9 | Male | DIPG | IV | Biopsy | 0.9 | Died | *H3F3A* | WT | p.R206H |
| **HSJD-DIPG-14A^2^** | DIPG | Pons | 8 | Female | DIPG | - | Biopsy | - | Died | *H3F3A* | WT | WT |
| **ICR-B117^3^** | DIPG | Pons | 7 | Male | DIPG | IV | Biopsy | - | Died | *H3F3A* | p.R273H | WT |
| **SU-DIPG-IV^4^** | DIPG | Pons | 2 | Female | DIPG | IV | Autopsy | 8.0 | Died | *HIST1H3B* | p.R273H | p.G328V |
| **SU-DIPG-VI^4^** | DIPG | Pons | 7 | Female | DIPG | III | Autopsy | 6.0 | Died | *H3F3A* | p.E198 | WT |

1.Vinci M,el al., Nat Med. 2018 Aug;24(8):1204-1215. PMID: 29967352.

2.Deligne C, et al., Fluids Barriers CNS. 2020 Jun 2;17(1):37. PMID: 32487241.

3. Mackay A, et al., Cancer Cell. 2018 May 14;33(5):829-842.e5. PMID: 29763623.

4. Nagaraja S, et al., Cancer Cell. 2017 May 8;31(5):635-652.e6. PMID: 28434841.

* Note HSJD-DIPG-007 and -014A are p53 wild-type by sequencing, the presence of PPM1D mutations may impair functional p53 activity.

**Supplementary Table 2. BH3 mimetics used in this study.**

| **Compound** | **Inhibitory profile**  **(K_i_ cell free assays)** | | | | **Clinical trial status** | **Example clinical trial** | **Achievable non-toxic C_max_ (nM)** |
| --- | --- | --- | --- | --- | --- | --- | --- |
|  | **Bcl-2** | **Bcl-xL** | **Bcl-w** | **Mcl-1** |  |  |  |
| **Venetoclax** | <0.01 | 48 | 245 | >440 | MHRA approved | NCT04401748 | 2533.4 ± 1612 |
| **Navitoclax** | <1 | <0.05 | <1 | 550  (+/-40) | Phase III | NCT02591095 | 6607 ± 3262 |
| **Obatoclax** | 0.2 | 1-7 | 1-7 | 1-7 | Phase I | NCT00600964 | 7-38 |
| **A-1331852** | 6 | <0.01 | 4 | 142 | Preclinical | n.a | n.a. |
| **A-1155463** | 74 | <0.01 | 8 | >444 | Preclinical | n.a | n.a |
| **A-1210477** | 132 | >660 | 2,280 | <0.5 | preclinical | n.a | n.a |

**Supplementary Table 3. Primary antibodies used in this study.**

| **Antibody Application** | **Target** | **Host Species** | **Clonality** | **Clone** | **Company** | **Catalogue Number** | **Dilution** | **Antigen Retrieval Method** |
| --- | --- | --- | --- | --- | --- | --- | --- | --- |
| **Primary** | Human nuclei antigen | Mouse | Monoclonal | N/A | Millipore | MAB1281 | 1:500 | Tris- EDTA pH9.0 |
| **Primary** | CX3CL1 | Rabbit | Monoclonal | N/A | R&D | AF472 | 1:1000 | Tris- EDTA pH9.0 |
| **Primary** | CRYAB | Mouse | Monoclonal | 1B6.1-3G4 | Abcam | R ab13496 | 1:300 | Tris- EDTA pH9.0 |
| **Primary** | Ki67 | Rabbit | Monoclonal | SP6 | Abcam | ab16667 | 1:100 | Tris- EDTA pH9.0 |
| **Primary** | P21 | Rabbit | Polyclonal | M-19 | Cell Signaling  Technology | #5487 | 1:400 | Tris- EDTA pH9.0 |
| **Primary** | P57 | Mouse | Monoclonal | 10B1 | Cell Signaling  Technology | #2557 | 1:200 | Tris- EDTA pH9.0 |
| **Primary** | P16 | Mouse | Monoclonal | N/A | Santa Cruz | Sc-56330 | 1:100 | Tris- EDTA pH9.0 |
| **Primary** | P27 | Mouse | Monoclonal | VE1 | Cell Signaling  Technology | E19290 | 1:50 | Tris- EDTA pH9.0 |
| **Primary** | pRB (Ser807/811) | Rabbit | Polyclonal | N/A | Cell Signaling  Technology | 9308 | 1:250 | Tris- EDTA pH9.0 |
| **Primary** | CCL2 | Rabbit | Monoclonal | N/A | R&D | MAB679R | 1:1000 | Tris- EDTA pH9.0 |
| **Primary** | IL1B | Rabbit | Monoclonal | N/A | R&D | MAB201 | 1:200 | Tris- EDTA pH9.0 |
| **Primary** | IL6 | Rabbit | Monoclonal | N/A | R&D | AF-206-NA | 1:1000 | Tris- EDTA pH9.0 |
| **Primary** | H3K27M | Rabbit | Polyclonal | N/A | Abcam | Ab19061 | 1:500 | Tris- EDTA pH9.0 |

**Supplementary Table 4. Secondary antibodies used in this study.**

| **Antibody Application** | **Target Species** | **Host Species** | **Clonality** | **Company** | **Catalogue Number** | **Dilution** | **Conjugate** |
| --- | --- | --- | --- | --- | --- | --- | --- |
| **Secondary** | Mouse | Goat | Polyclonal | Thermo Fisher | A11001 | 1:250 | Alexa Fluor® 488 |
| **Secondary** | Mouse | Goat | Polyclonal | Thermo Fisher | A11004 | 1:250 | Alexa Fluor® 568 |
| **Secondary** | Rabbit | Goat | Polyclonal | Thermo Fisher | A11008 | 1:250 | Alexa Fluor® 488 |
| **Secondary** | Rabbit | Goat | Polyclonal | Thermo Fisher | A11036 | 1:250 | Alexa Fluor® 568 |
| **Secondary** | Goat | Chicken | Polyclonal | Thermo Fisher | A21467 | 1:250 | Alexa Fluor® 488 |
| **Secondary** | Mouse | Goat | Polyclonal | Dako | E0433 | 1:250 | Biotin |
| **Secondary** | Rabbit | Goat | Polyclonal | Dako | E0432 | 1:250 | Biotin |
| **Secondary** | Mouse | Goat | Polyclonal | Dako | P0447 | 1:250 | Horseradish peroxidase (HRP) |
